# Factors associated with noncommunicable disease among adults in Mecha district, Ethiopia: A case control study

**DOI:** 10.1371/journal.pone.0216446

**Published:** 2019-05-29

**Authors:** Yeshalem Mulugeta Demilew, Belet Sewasew Firew

**Affiliations:** 1 School of Public Health, College of Medicine and Health Sciences, Bahir Dar University, Bahir Dar, Ethiopia; 2 School of Medicine, College of Medicine and Health Sciences, Bahir Dar University, Bahir Dar, Ethiopia; University of British Columbia, CANADA

## Abstract

**Background:**

In Ethiopia, the incidence and prevalence of noncommunicable diseases are rising. Within the country, the magnitude of these diseases varies from region to region. However, information about factors associated with noncommunicable disease is limited in the study area. Therefore, the objective of this study was to identify factors associated with noncommunicable disease among adults in Mecha district.

**Methods:**

Community-based case-control study was carried out among 728 cases and 2907 controls from February1-August 30/2017. The study participants were chosen using a multi-stage sampling technique. Data were collected using structured questionnaire. Fasting blood glucose level was measured in the morning after 8hours of fasting. Statistical Package for Social Science (SPSS) version 20 software was used to enter and analyze data. Crude and adjusted Odds ratios were done for each explanatory variable at 95% confidence level.

**Results:**

The likelihood of developing noncommunicable disease was higher among participants who drank alcohol [AOR = 1.72, 95% CI: (1.3, 2.1)] and coffee [AOR = 4.54, 95% CI: (3.4, 5.9)], did not take vegetables [AOR = 2. 30, 95% CI: (1.6, 3.1)] and fruits [AOR = 2.04, 95% CI: (1.4, 2.9)], took packed oil [AOR = 2.35, 95% CI: (1.7, 3.1)], overweight or obesity [AOR = 2.23, 95% CI: (1.3, 3.8)] and physically inactive [AOR = 1.71, 95% CI: (1.2, 2.4)].

**Conclusion:**

Of those assessed, the main factors associated with noncommunicable disease were drinking alcohol and coffee, not taking vegetables and fruits, taking packed oil, being overweight and physically inactive. Thus, the finding suggests changing the dietary habit of the community to increase consumption of fruits and vegetables, use of unsaturated fat for cooking, to avoid consumption of alcohol and to decrease taking coffee, to do physical activity and weight reduction.

## Introduction

Noncommunicable diseases (NCDs) are non-contagious diseases with long duration. It includes heart disease, stroke, cancer, asthma, diabetes mellitus, chronic kidney disease, arthritis, osteoporosis and cataracts[1]. The prevalence of NCDs increases throughout the world. It leads to 47% of the disease burden and 63% of all mortalities[2]. Of which, 80% of mortalities occur in developing countries, and the majority of deaths are premature[2–4].

Further, by the year 2020, global anticipated NCDs burden will rise to 80% and the majority of deaths (70%) will occur in low and middle-income countries[4,5]. Similarly, the magnitude of NCDs is increasing in Ethiopia[1,6]. Hypertension and diabetes mellitus (DM) are the two most common and easily diagnosed forms of NCDs[7,8]. There are one billion hypertensive cases worldwide[7,9]. Of which one in three patients live in developing countries [2,5,10]. In Ethiopia too, the magnitude of hypertension increased from 18.8% in 2010 [11] to 27.9% in 2015[12].

Hypertension is a significant risk factor for illness and death due to myocardial infarction, stroke, atherosclerosis, aortic aneurysm, heart failure, peripheral artery disease, and end-stage renal disease[13]. It contributes to13% of global mortality and 7% of global disability-adjusted life years[14].

In 2013, globally, 8.3% of adults (382 million people) had diabetes. Among them, 80% of diabetes cases live in developing countries[15]. Likewise, 6.5% of Ethiopian adults had DM [16]. Diabetes mellitus is the major risk factor for coronary artery disease, peripheral arterial disease, stroke, cardiomyopathy, congestive heart failure, diabetic nephropathy, neuropathy, and retinopathy[17].

Several Scholars reported the association between increased age, physical inactivity, family history of hypertension, having diabetes, use of alcohol, central obesity and hypertension [18,19]. Whereas, some studies showed lack of association between khat chewing, cigarette smoking, use of coffee, sex, physical inactivity, alcohol intake and hypertension [20,21]. Physical inactivity, overweight or obesity, having hypertension, increased age, inadequate intake of fruits and vegetables, low education, married or divorced, jobless/housewives, current smokers and having low salaries are risk factors associated with diabetes mellitus [22,23].

The risk factors of diabetes mellitus and hypertension are similar [6,24,25]. The presence of diabetes mellitus predisposed to hypertension and vice versa. In the literature, contradictory ideas were reported on factors associated with NCDs. Therefore, the aim of this study was to identify factors associated with NCDs in Mecha district.

## Methods

### Study area

This study was carried out in Mecha district. The district is one of the 15 districts in West Gojjam Zone, Amhara Region, Ethiopia with a total population of 3,720,716. Of the total population, about 1,837,289 are females. The district is located at 35 kilometers Southwest of Bahir Dar on the main road of Bahir Dar to Addis Ababa. It has 40 rural and 3 urban Kebeles with a total of one hospital, ten health centers, and forty health posts.

### Study design and period

This study used a case-control study design to assess the risk factors for noncommunicable diseases. In this study, known diabetes or hypertensive patients on medication and patients who diagnosed as new diabetes or hypertensive case during community-based screening by Mecha Demographic Surveillance and Field Research Center, Bahir Dar University were recruited as a case from February 01/2017-August 30/2017. Appropriate controls with similar age were recruited as control.

### Source population

The study participants were all adults age 30 years old or above in Mecha district. Adults who had hypertension and/or diabetes mellitus were considered as a case while adults who had no hypertension or diabetes mellitus were categorized in the control group.

### Sample size

A two population proportion formula using Epi-info software version 7.2.1.0 was used to determine the sample size of this study. The following assumptions were considered to estimate the required sample size; the expected proportion of exposure among cases was 50% and 40% among controls since there was no study conducted on the determinants of NCDs. Using these values with confidence level of 95%, power of 90%, a case to control ratio of 1:4, odds ratio of 1.5, considering a 10% contingency for non-response rate and design effect 2, a sample size of **742** cases with **2946** controls was required.

### Sampling procedure

The study participants were chosen using multi-stage sampling technique. First, the district was stratified into urban and rural Kebeles (the smallest administrative unite in Ethiopia). Next, one urban and twelve rural Kebeles were selected by simple random sampling (lottery) method. Screening for DM and hypertension was done by Mecha Demographic Surveillance and Field Research Center, Bahir Dar University through house-to-house visit to identify cases in selected Kebeles. List of all cases in selected Kebeles was used to select cases.

The sample size was allocated to each Kebele considering proportional to the size assignment based on the number of cases in selected Kebeles. Finally, both cases and controls were again selected by simple random sampling method using list of cases and controls in the research center. During sample selection, if more than one case or control were selected in the same household lottery method was used to select one study participant in the household.

Noncommunicable disease (diabetes mellitus and/or hypertension) was the dependent variable. In this study, noncommunicable disease includes DM and hypertension. Diabetes mellitus was defined as a fasting capillary whole blood glucose value ≥126mg/dl or on treatment with anti-diabetes medication. Hypertension was defined when the average systolic blood pressure readings ≥140 mmHg and/or diastolic blood pressure readings ≥90 mmHg or on treatment with anti-hypertensive medication. Body mass index < 18.5kg/m^2^ was considered as energy deficient, BMI 18.5–24.99 kg/m^2^ labeled as normal, BMI 25–29.99kg/m^2^ was considered overweight and BMI ≥30 was taken as obesity.

Since, Tella (Ethiopian drink), Areki and beer are the main types of alcoholic drinks in the study area; alcohol consumption was defined using these three alcohols. Alcohol consumption was defined as taking more than a glass of Tella and/or a small cup of Areki and/or a bottle of beer more than three times per week for females and more than two glass of Tella and/or small cup of Areki and/or more than two bottles of beer more than three times per week for males.

In this study packed oil is “Hayat” or “OKI” oil which is packed in a jar.

Physically active: a person performs ≥ 150 minutes of moderate-intensity aerobic exercise or at least ≥75 minutes of vigorous-intensity aerobic exercise per week or an equivalent combination of the two activities. Moderate physical activity: performing up to 150 minutes of moderate-intensity exercise per week or up to 75 minutes of vigorous-intensity exercise per week or stair-climbing, mopping a floor, general gardening, moderate farm activities, otherwise physically inactive[26].

### Data collection

Interviewers administered structured questionnaires. The questionnaire was adapted from the WHO STEPwise approach for noncommunicable diseases in developing countries [27,28]. The questionnaire was customized according to the local context and study objective. Data were collected through house-to-house visit. The data collection team composed of three laboratory technicians who measured fasting blood glucose level, thirteen diploma nurses who collect data, and four health officers’ who do supervision. Two days training on interviewing techniques, anthropometric measurements and ethical issues were given to the data collection team. Pre-test was done before the actual data collection using 5% of respondents from a similar setting. Based on the pretest, questions were revised.

#### Measurements

Fasting blood glucose level was measured as per WHO recommendation [29]. A peripheral blood sample was collected after 8 hours of fasting early in the morning before participants took their breakfast. Blood sample was taken by finger puncture, using proper sanitation and infection prevention techniques. The collected blood sample was used to determine participants’ fasting blood glucose level. The WHO and International Diabetes Association criteria was used to classify fasting blood glucose level [29]. When fasting blood glucose level was ≥126mg/dl, a person was considered as has diabetes mellitus whereas when fasting blood glucose level was <126mg/dl a person was labeled as has no diabetes mellitus.

Weight and height were measured. Weighing scale (SECA Germany) was used to measure weight. During weight measurement, each participant wears light cloth. Before each measurement the weighing scale was calibrated to zero and functionality was checked using a well-known weight object. Weight was measured to the nearest 0.1kg and two measurements were taken from each participant and the average was taken during the analysis. Height was measured using a stadiometer. During height measurement, participants’ stood keeping the normal anatomical position, and heels, buttock, shoulder, and back of the head touching the measuring board. Height was recorded to the nearest 0.5 cm and two measures were taken and the average was considered during the analysis. Body mass index (BMI) was calculated as the ratio of weight in kilogram to the square of height in meter [30].

Blood pressure (BP) was measured using mercury sphygmomanometer with participants sitting after resting for at least five minutes. Instruction was given to avoid talking and to breathe normally during the time of measurement. Two additional blood pressure measurements were taken with fifteen minutes elapsing between successive measurements. In accordance with the WHO recommendation, the mean systolic and diastolic BP was considered for analyses.

### Data quality control issues

To assure the quality of data, properly designed, pretested data collection instruments were used. Two days training was given for data collectors, laboratory technicians and supervisors. Supervisors and investigators closely followed data collection process. The completeness of questionnaires was checked by supervisors and investigators on each day of data collection. After checking for consistency and completeness, supervisors submit the filled questionnaires to the investigators. The collected data were double entered by investigators to verify whether the data were properly entered or not by the data clerk.

### Data management and analysis

Data were checked for completeness. Completed questionnaires were coded and entered into SPSS version 20.0 software for analysis. A frequency of each variable was calculated to check for accuracy, outliers, consistency and missed values. Variables entered into the first unadjusted model were age, marital status, educational and occupational status, drinking of alcohol and coffee, physical activity and BMI.Crude and adjusted Odds ratios were done for each explanatory variable at 95% confidence level. Variables with p-value ≤0.2 were taken into the final adjusted model. The model was adjusted for age and sex. Variables with p-value less than 0.05 were considered as statistical significant in the final model.

### Ethical consideration

This study was approved by Institutional Review Board of Bahir Dar University. Letter of permission was secured from zonal, district and Kebele administrators. Written consent (fingerprint for those who cannot read and write) was taken from each study participant. Each questionnaire was number-coded without any personal identification to keep privacy and confidentiality throughout the study period. Nutrition education was given for cases and controls. Cases who did not start treatment were referred to Merawi hospital to get treatment and follow up service after giving nutrition education.

## Results

### Socio-demographic characteristics of the study participants

Among **742** cases and **2946** controls invited, 728 cases and 2907 controls participated in the study and gave complete information; make a response rate of 98.1% and 98.6% respectively. About 27.7% of cases and 59.9% of controls were age below 45years old, whereas 23.4% of cases and 8.2% of controls were above 64years old. More than half (56.9%) of cases and 51.5% of controls were females.

Nearly three in four (72.4%) cases and two in three controls were urban residents. Almost all cases and controls (98.8%) were Orthodox Christian in religion. Regarding their ethnicity, 99.1% of cases and 99.2% of controls were Amhara in ethnicity. Three fourth of cases (77.5%) and controls (75.3%) had no formal education and 13.6% of cases and 26.6% of controls were farmers (Table 1).

**Table 1:** Socio-demographic characteristics of the study participants in Mecha district, Ethiopia, 2017.

Variables	Total (3635)	Cases (n = 728)	Controls (n = 2907)	P-value
Age (years)				
**30–44**	1943(53.5)	202(27.7)	1741(59.9)	
**45–64**	1284(35.3)	356(48.9)	928(31.9)	
**≥65**	408(11.2)	170(23.4)	238(8.2)	<0.001
Sex				
**Male**	1722(47.3)	314(43.1)	1408(48.4)	0.01
**Female**	1913(52.6)	414(56.9)	1499(51.6)	
Residence				
**Urban**	2442(67.2)	527(72.4)	1915(65.8)	0.001
**Rural**	1193(32.8)	201(27.6)	992(34.1)	
Religion				
**Orthodox**	3591(98.8)	719(98.8)	2872(98.8)	0.94
Others*	44(1.2)	9(1.2)	35(1.2)	
Ethnicity				
**Amhara**	3602(99.1)	718(98.6)	2884(99.2)	0.13
Others**	33(0.9)	10(1.4)	23(0.8)	
Educational status				
**No formal education**	2753(75.7)	564(77.5)	2189(75.3)	0.13
**Primary education**	254(7.0)	37(5.1)	217(7.5)	
**Above primary education**	628(17.3)	127(17.4)	501(17.2)	
Occupational status				
**Housewife**	1310(36.0)	325(44.6)	985(33.9)	<0.001
**Farmer**	874(24.1)	99(13.6)	775(26.6)	
**Government employee**	399(11)	100(13.8)	299(10.3)	
**Private employee**	1052(28.9)	204(28.0)	848(29.2)	
Marital status				
**Married**	2745(75.5)	519(71.3)	2226(76.6)	0.003
Others***	890(24.5)	209(28.7)	681(23.4)	

Others* = Muslim & protestant

others** = Agew, Tigrie & Oromo

Others*** = Unmarried, divorced & widowed

### Behavioral risk factors to noncommunicable disease among adults

More cases (76%) than controls (66.9%) drank alcohol. Among alcohol users, 43.8% of cases and 38.7% of controls drank Tella, Areki and beer frequently. More than half (57.3%) cases and 63.0% of controls drank alcohol more than 10 years. More cases (89.8%) than controls (67.5%) drank coffee. Only few, 0.7% cases and 0.2% controls had previous history of smoking cigarettes but no smoker in both cases and controls during the time of data collection. About 2.7% of cases and 2.3% of controls chewed Khat in their lifetime but no one chewed Khat during the time of data collection (Table 2).

**Table 2:** Behavioral risk factors of noncommunicable disease among adults in Mecha district, Ethiopia, 2017.

Variables	Cases (n = 728)	Controls (n = 2907)	P-value
**Ever smoke cigarettes**			
Yes	5(0.7)	7(0.2)	0.06
No	723(99.3)	2900(99.8)	
**Took vegetables**			
Occasionally (≤1times weekly)	71(9.8)	710(24.4)	<0.001
No	657(90.2)	2197(75.6)	
**Took fruits**			
Occasionally(≤1times weekly)	43(5.9)	674(23.2)	<0.001
No	685(94.1)	2233(76.8)	
**Oil**			
Packed	644(88.5)	2353(80.9)	<0.001
Niger seed	84(11.5)	555(19.1)	
**Ever chewed Khat**			
Yes	20(2.7)	67(2.3)	
No	708(97.3)	2840(97.7)	0.48
**Body Mass index**			
<18.5kg/m^2^	76(10.4)	421(14.5)	<0.001
18.5–24.99 kg/m^2^	617(84.8)	2402(82.6)	
≥25kg/m^2^	35(4.8)	84(2.9)	
**Drank alcohol**			
**Yes**	553(76.0)	1944(66.9)	<0.001
**No**	175(24.0)	963(33.1)	
**Type of alcohol (n = 553 cases & 1944 controls)**			
Tella (local beer)	311(56.2)	1192(61.3)	0.03
Areki, Tella and beer	242(43.8)	752(38.7)	
**Duration of drinking alcohol (years) (n = 553 cases & 1944 controls)**			
<10	38(6.9)	33(1.7)	0.008
10–19	198(35.8)	686(35.3)	
≥20	317(57.3)	1225(63.0)	
**Drank coffee**			
Yes	654(89.8)	1963(67.5)	<0.001
No	74(10.2)	944(32.5)	
**Physical activity**			
Low	267(36.7)	402(13.8)	<0.001
Moderate	224(30.8)	1339(46.1)	
High	237(32.5)	1166(40.1)	

More controls (24.4%) ate vegetables occasionally (at least once per week) compared with cases (9.8%). Similarly, 23.2% of controls and 5.9% of cases took fruits one or less times per week. Majority of cases (88.5%) and controls (80.9%) used packed oil for cooking. More cases (4.8%) compared with controls (2.9%) were overweight or obese. Moreover, 36.7% of cases and 13.8% of controls did low level of physical activity (Table 2).

### Factors associated with noncommunicable disease

Not taking fruits and vegetables, female sex, being urban dweller, drinking alcohol and coffee, overweight or obesity, low level of physical activity, not married, eating packed oil, being housewives/government or private employee and age over 45 years old were factors associated with noncommunicable disease in the bi-variable logistic regression analysis (Table 3).

**Table 3:** Factors associated with noncommunicable disease in Mecha district, Ethiopia, 2017.

Variables	Noncommunicable disease		COR ((95%CI)	AOR(95%CI)
	Yes	No		
**Sex**				
Male	313(43.0)	1408(44.4)	1.00	1.00
Female	414(57.0)	1499(51.7)	1.23(1.1,1.4)	1.15(0.9,1.4)
**Residence**				
Urban	527(72.4)	1915(65.8)	1.35(1.1,1.6)	1.27(1.0,1.6)
Rural	201(27.6)	992(34.1)	1.00	1.00
**Age (years)**				
30–44	202(27.7)	1741(59.9)	1.00	1.00
45–64	356(48.9)	928(31.9)	3.30(2.7,4.0)	3.29(2.6,4.1)
≥65	170(23.4)	238(8.2)	6.15(4.8,7.8)	3.78(2.4,5.7)
**Drink Alcohol**				
Yes	553(76.0)	1944(66.9)	1.56(1.3,1.9)	1.72(1.3,2.1)
No	175(24.0)	963(33.1)	1.00	1.00
**Drink coffee**				
Yes	654(89.8)	1963(67.5)	4.25(3.3,5.5)	4.54(3.4,5.9)
No	74(10.2)	944(32.5)	1.00	1.00
**Physical activity**				
Low	267(36.7)	402(13.8)	3.26(2.6,4.0)	1.71(1.2,2.4)
Moderate	224(30.8)	1339(46.1)	0.82(0.7,1.0)	0.85(0.6,1.0)
High	237(32.5)	1166(40.1)	1.00	1.00
**Marital status**				
Married	519(71.3)	2226(76.6)		1.00
Unmarried/widowed/divorced	209(28.7)	681(23.4)	1.31(1.1,1.6)	1.22(0.9,1.5)
**Took vegetables**				
Occasionally	71(9.8)	710(24.4)	1.00	1.00
No	657(90.2)	2197(75.6)	2.99(2.3,3.9)	2.30(1.6,3.1)
**Took fruits**				
Occasionally	43(5.9)	674(23.2)	1.00	1.00
No	685(94.1)	2233(76.8)	4.80(3.5,6.6)	2.04(1.4,2.9)
**Oil**				
Packed	644(88.5)	2353(80.9)	1.80(1.4,2.3)	2.35(1.7,3.1)
Niger seed	84(11.5)	555(19.1)	1.00	1.00
**Occupational status**				
Farmer	99(13.6)	775(26.6)	1.00	1.00
Housewife	325(44.7)	985(33.9)	2.58(2.0,3.3)	2.74(2.0,3.6)
P/employee	204(28.0)	848(29.2)	1.88(1.4,2.4)	2.31(1.6,3.1)
G/employee	100(13.7)	299(10.3)	2.61(1.9,3.6)	3.43(2.3,4.9)
**Body mass index**				
<18.5kg/m^2^	76(10.4)	421(14.5)	0.70(0.5,0.9)	1.30(0.9,1.7)
18.5–24.99 kg/m^2^	617(84.8)	2402(82.6)		1.00
≥25kg/m^2^	35(4.8)	84(2.9)	1.8(1.2,2.8)	2.23(1.3,3.8)

COR- Crude odds ration; AOR–Adjusted odds ratio, CI- Confidence interval

The multivariable logistic regression analysis revealed the association between noncommunicable disease and not taking fruits and vegetables, drinking alcohol and coffee, overweight or obesity, low level of physical activity, eating packed oil, housewives/government or private employee and age over 45 years old (Table 3).

Participants with age 45–64 years were 3.29 times [AOR = 3.29, 95% CI: (2.6, 4.1)] and whose age above 64 years were 3.78 times [AOR = 3.78, 95% CI: (2.4, 5.7)] more likely to have NCDs than respondents whose age less than 45 years old. The study participants who drank alcohol were 1.72 times prone to have NCDs than participants who did not drink alcohol [AOR = 1.72, 95% CI: (1.3, 2.1)]. Likewise, respondents who drank coffee were 4.54 times more likely to have NCDs compared with their counterparts [AOR = 4.54, 95% CI: (3.4, 5.9)] (Table 3).

The odds of having NCDs was 1.71 times higher among the study participants who did low level of physical activity than respondents who did high level of physical activity [AOR = 1.71, 95% CI: (1.2, 2.4)]. Another predictor for NCDs was avoiding vegetables from the diet; participants who did not eat vegetables were 2.3 times more likely to have NCDs than participants who took vegetables occasionally [AOR = 2.30, 95% CI: (1.6, 3.1)]. Similarly, the odd of having NCDs was 2 times higher among participants who did not eat fruits than respondents who ate fruits occasionally [AOR = 2.04, 95% CI: (1.4, 2.9)] (Table 3).

Eating packed oil also showed statistically significant association with NCDs, participants who used packed oil for food preparation were 2.3 times prone to have NCDs than respondents who used Niger seed oil for food [AOR = 2.35, 95% CI: (1.7, 3.1)]. Occupational status of the study participants had association with NCDs, housewives were 2.74 times [AOR = 2.74, 95% CI: (2.0, 3.6)], private employees were 2.3 times [AOR = 2.31, 95% CI: (1.6, 3.1)] and government employees were 3.4 times [AOR = 3.43, 95% CI: (2.3, 4.9)] more likely to have NCDs than farmers. Overweight or obesity were another predictors for NCDs; overweight or obese participants were 2.23 times more likely to have NCDs compared with normal nourished participants [AOR = 2.23, 95% CI: (1.3, 3.8)] (Table 3).

## Discussions

The aim of this study was to assess factors associated with NCDs among adults in Mecha district. In this study age, occupation, drinking alcohol, using packed oil, drinking coffee, physical inactivity and not taking vegetables and fruits were predictors of NCDs.

When age increases the probability of having NCDs was also increased. This finding was similar with other study findings in Dabat Ethiopia [31], Afghanistan [32], Uganda [33] and Saudi [34]. The possible justification might be due to progressive reduction of the strength of musculature with age which causes muscular atrophy. Moreover, decreased economic productivity and social isolation lead to psychological problems. All these, in turn, predispose to NCDs.

Drinking coffee appeared to be the strongest predictor in determining the probability of having NCDs. This study finding is in agreement with the study finding of SUN Project [35] but the finding isn’t consistent with a study finding in Brazil [36]. The reason for this discrepancy might be the habit of drinking coffee with salt and sugar in Ethiopia. Additionally, Ethiopian people took multiple cups of coffee more than once a day; this persistently increases plasma level of stress hormones which in turn increase the probability of having NCDs.

In this study, alcohol was found to be positively associated with NCDs. Similar studies showed the association between alcohol and NCDs [37,38]. The possible explanation might be due to the role of alcohol in increasing triglyceride level and its effect in decreasing body sensitivity to insulin.

World Health Organization and Food and Agriculture Organization have suggested consumption of 400g (five serving) of fruits and vegetables per day for the prevention of noncommunicable disease [39]. However, in this study, only few respondents took fruits and vegetables ≤ once a week but no one took fruits or vegetables daily.

Study participants who did not take vegetables and fruits were more likely to have NCDs. This finding was in agreement with several previous study findings [40–43]. This might be due to the benefit of adequate consumption of fruits and vegetables to provide dietary fibre and other essential nutrients that lower the risk of NCDs. Additionally, fruits and vegetables have significant effect to lower low-density lipoprotein level.

Scholars recommend replacing trans-fat by unsaturated fat due to a high probability of developing NCDs among trans-fat and saturated fat users compared with unsaturated fat consumers[44]. In this study, participants who used packed oil for cooking were more likely to have NCDs compared with participants who used niger seed oil. This is because hydrogenated vegetable oils increase abdominal fat distribution and the risk of NCDs by increase low-density lipoprotein level and lower high-density lipoprotein level. Low-density lipoproteins stay in the bloodstream for longer time, resulting in higher fasting insulin concentrations and lower insulin sensitivity.

Housewives and private or government employees were more likely to develop NCDs than farmers. This finding was supported by the study finding in China in which not being involved in farm work were found to be positively associated with NCDs [31,45]. This might be due to the probability of having low level of physical activity among housewives and employees compared with farmers who engaged in intense agricultural activities.

Obesity has been identified as a strong predictor for hypertension and diabetes [46]. Obese individuals were more likely to have hypertension and diabetes mellitus compared with their counterparts. This finding was similar with previous study findings [47,48]. This might be due to the fact that obese people have excessive secretion of nonesterified fatty acids from adipose tissue which cause insulin resistance and β-cell dysfunction. Additionally, obesity increased blood cholesterol level and vascular wall thickness.

The likelihood of having NCDs was higher among individuals who did low level of physical activity compared with their counterparts. This finding was in line with the study findings in Dabat, Ethiopia,[31], Jos, Nigeria [13] and India [47]. Low level of physical activity increase an overall positive energy balance, which directly contributes for the development of NCDs. Whereas, lack of association was observed between physical inactivity and NCDs in a study done among physicians in Central Saudi Arabia [49]. This might be due to increase in oxidative stress, endothelial dysfunction, body mass, rennin-angiotensin system activity, sympathetic activity and insulin resistance in sedentary lifestyle.

### Strength and limitation of the study

Being a community-based study which can truthfully describe the general population is the strength of this study. However, case-control studies aren’t able to establish causal relationship.

### Conclusion and recommendation

Of the variables we examined, **t**he predominant factors associated with noncommunicable disease were drinking alcohol and coffee, did not take vegetables and fruits, taking packed oil, overweight and low level of physical activity. Thus, the finding suggests changing dietary habit of the community to increase consumption of fruits and vegetables, use of unsaturated fat for cooking, to avoid consumption of alcohol and coffee, to do physical activity and weight reduction.

## Abbreviations

AOR: Adjusted Odds Ratio
BMI: Body Mass Index
CI: Confidence Interval
COR: Crude Odds Ratio
DM: Diabetes Mellitus
NCDs: Non Communicable Diseases
WHO: World Health Organization
